# Inflammatory Indexes as Prognostic Factors of Survival in Geriatric Patients with Hepatocellular Carcinoma: A Case Control Study of Eight Slovak Centers

**DOI:** 10.3390/jcm11144183

**Published:** 2022-07-19

**Authors:** Dominik Safcak, Sylvia Drazilova, Jakub Gazda, Igor Andrasina, Svetlana Adamcova-Selcanova, Lea Balazova, Radovan Barila, Michal Mego, Marek Rac, Lubomir Skladany, Miroslav Zigrai, Martin Janicko, Peter Jarcuska

**Affiliations:** 1 Department of Radiotherapy and Oncology, East Slovakia Institute of Oncology, Rastislavova 43, 041 91 Kosice, Slovakia; safcak@vou.sk (D.S.); andrasina@vou.sk (I.A.); lea.balazova@gmail.com (L.B.); 2 Internal Medicine Department, Hospital Poprad a.s., Banicka 803, 058 01 Poprad, Slovakia; 3 2nd Department of Internal Medicine, P. J. Safarik University, Faculty of Medicine and L. Pasteur University Hospital, Trieda SNP 1, 040 11 Kosice, Slovakia; jakub.gazda@upjs.sk (J.G.); martin.janicko@upjs.sk (M.J.); peter.jarcuska@upjs.sk (P.J.); 4 2nd Department of Internal Medicine, HEGITO, F. D. Roosevelt University Hospital, Namestie L Svobodu 1, 975 17 Banska Bystrica, Slovakia; sselcanova@gmail.com (S.A.-S.); lubomir.skladany@gmail.com (L.S.); 5 Oncological Cluster, Stefan Kukura Hospital in Michalovce, Spitalska Ulica 2, 071 01 Michalovce, Slovakia; radovan.barila@gmail.com; 6 2nd Department of Oncology, Faculty of Medicine, Comenius University and National Oncology Institute of Slovakia, Klenova 1, 833 10 Bratislava, Slovakia; misomego@gmail.com; 7 Department of Internal Medicine, Teaching Hospital Nitra, Spitalska 6, 949 01 Nitra, Slovakia; marek.rac@fnnitra.sk; 8 1st Department of Internal Medicine, Ladislav Derer University Hospital in Bratislava, Limbova 5, 833 05 Bratislava-Kramare, Slovakia; miroslav.zigrai@szu.sk

**Keywords:** hepatocellular cancer, geriatric patients, overall survival, progression-free survival

## Abstract

Background and Aims: Hepatocellular cancer (HCC) often occurs in geriatric patients. The aim of our study was to compare overall survival and progression-free survival between geriatric patients (>75 years) and patients younger than 75 years and to identify predictive factors of survival in geriatric patients with HCC. Material and Methods: We performed a retrospective analysis of patients with HCC diagnosed in Slovakia between 2010–2016. Cases (HCC patients ≥75 years) were matched to controls (HCC patients <74 years) based on the propensity score (gender, BCLC stage and the first-line treatment). Results: We included 148 patients (84 men, 57%) with HCC. There were no differences between cases and controls in the baseline characteristics. The overall survival in geriatric patients with HCC was comparable to younger controls (*p* = 0.42). The one-, two-, and three-year overall survival was 42% and 31%, 19% and 12%, and 12% and 9% in geriatric patients and controls, respectively (*p* = 0.2, 0.4, 0.8). Similarly, there was no difference in the one- and two-year progression-free survival: 28% and 18% vs. 10% and 7% in geriatric HCC patients and controls, respectively (*p* = 0.2, 1, -). There was no case–control difference between geriatric HCC patients and younger HCC controls in the overall survival in the subpopulation of patients with no known comorbidities (*p* = 0.5), one and two comorbidities (*p* = 0.49), and three or more comorbidities (*p* = 0.39). Log (CRP), log (NLR), log (PLR), and log (SII) were all associated with the three-year survival in geriatric HCC patients in simple logistic regression analyses. However, this time, only log (NLR) remained associated even after controlling for the age and BCLC confounding (OR 5.32, 95% CI 1.43–28.85). Conclusions. We found no differences in overall survival and progression-free survival between older and younger HCC patients. Parameters of subclinical inflammation predict prognosis in geriatric patients with HCC. A limitation of the study is small number of the treated patients; therefore, further investigation is warranted.

## 1. Introduction

Liver cancer was the sixth most frequently diagnosed cancer and the third most common cause of cancer death worldwide in 2020. Incidence and mortality rates are 2–3 times greater in the male population, and the incidence rates are highest mainly in transitioning countries. Among all histological subtypes, hepatocellular carcinoma (HCC) represents 75–85% of all diagnosed cases. Most hepatocellular cancer cases are attributed to chronic liver disease resulting from hepatitis B virus (HBV) or hepatitis C virus (HCV) infection, alcohol abuse, non-alcoholic fatty liver disease (NAFLD), aflatoxin-contaminated food intake, and smoking. All of these risk factors vary by region [1].

Barcelona Clinic Liver Cancer is the most widely accepted staging system for providing prognostic information and guidance in therapeutic strategy for patients with HCC. (BCLC) According to the European Association for the Study of the Liver (EASL), the European Society for Medical Oncology (ESMO), the American Association for the study of the Liver Diseases (AASLD), and the American Society of Clinical Oncology (ASCO), curative treatment (including resection, transplantation, and radiofrequency ablation) is indicated for patients in stage BCLC 0 and A, palliative treatment (transarterial chemoembolization and systemic treatment with biological treatment and/or immunotherapy) is recommended for patients with stages BCLC B and C, and the best supportive care is reserved for subjects in stage BCLC D [2,3,4,5].

Data from developed countries (the UK, the USA, Canada, and Taiwan) show a trend of increasing incidence in the elderly population [6,7].

In addition, inflammatory responses have recently been shown to influence tumor prognosis by interfering with the tumor microenvironment [8]. Many studies show a negative impact of increased inflammatory indexes such as the neutrophil to lymphocyte ratio (NLR), platelet to lymphocyte ratio (PLR), and systemic immune-inflammation index (SII) on overall survival of treated patients with HCC [9,10,11,12,13,14].

The aim of our presented study was to compare overall survival in both age groups (elderly and younger patients) and to investigate the influence of inflammatory markers (CRP level, NLR, PLR, and SII), ALBI score, and number of comorbidities on survival parameters.

## 2. Materials and Methods

We performed a multicenter retrospective longitudinal case–control study of patients diagnosed with HCC at eight specialized centers in Slovakia during the period from 2010 to 2016 (Banska Bystrica, Bratislava (2), Kosice (2), Michalovce, Nitra, and Poprad). The inclusion criterion was the diagnosis of HCC consistent with the EASL-EORTC guidelines (HCC confirmed by either histopathological examination or magnetic resonance imaging). Patients with uncertain histology, combined histology with cholangiocellular carcinoma, or any concurrent malignancy were excluded from the study.

Initially, we screened all patients diagnosed with a malignant neoplasm of the liver and intrahepatic bile ducts (ICD-10 cm C22) and identified 483 patients with a diagnosis of hepatocellular carcinoma (ICD-10 cm C22.0). Among these subjects, we identified 74 cases aged 75 and older at the time of hepatocellular carcinoma presentation.

The Child–Pugh score was calculated to estimate cirrhosis severity, and performance status was evaluated using the Eastern Cooperative Oncology Group Scale. CT scans of the thorax, abdomen, and pelvis were used to identify potential extrahepatic spread. All centers used the Barcelona Clinic Liver Cancer (BCLC) staging system to guide the management of patients.

Case report forms (CRFs) included baseline blood test results, which were also later used to calculate the neutrophil-to-lymphocyte ratio (NLR) [15], the platelet-to-lymphocyte ratio (PLR) [16], systemic immune-inflammation index (SII) [17], the model for end-stage liver disease score (MELD) [18], and the albumin–bilirubin grade (ALBI) [19]. If any condition that could have influenced baseline values was present (acute infection, corticosteroid treatment, etc.), repeated analyses were performed after the restoration of that condition. The CRFs also included the date of death, extracted either from the patients’ medical records or from the database of the Slovak Health Care Surveillance Authority.

Comorbidities of interest included cardiovascular diseases (arterial hypertension, congestive heart failure, arterial fibrillation, ischemic heart disease, valve disorders) chronic kidney disease, chronic pulmonary diseases (chronic pulmonary obstructive disease, bronchial asthma, interstitial lung fibrosis), endocrine disorders (diabetes mellitus-including long-term related complications, hypothyroidism), gastroenterological disorders (inflammatory bowel disease, chronic pancreatitis, esophageal varices), and neurological disorders (peripheral exotoxic neuropathy and Parkinson’s disease), and were collected from CRFs’ medical history at the time of first presentation of hepatocellular carcinoma.

The study protocol was in accordance with the 1964 Declaration of Helsinki, its later amendments, and the principles of good clinical practice. The study protocol was approved by the Ethics Committee of East Slovakia Oncological Institute on 27 May 2021 (approval code, EK/2/05/2021). The committee waived the need for the patients’ informed consent due to the retrospective nature of the data collection and analysis and publication of only anonymous data.

## 3. Statistical Analyses

Data are presented as absolute counts and frequencies and medians and interquartile ranges (IQR). A Kaplan–Meier plot was used to describe the survival data graphically. The significance of differences in data distribution was tested using the Wilcoxon rank-sum test, Pearson’s Chi-squared test, Fisher’s exact test, or Log-rank test as appropriate. Simple logistic regression analyses were performed to analyze the association of baseline factors and the survival of patients, and multiple logistic regression analyses were later used to control for confounding effects of particular variables.

## 4. Results

**Figure 1:**
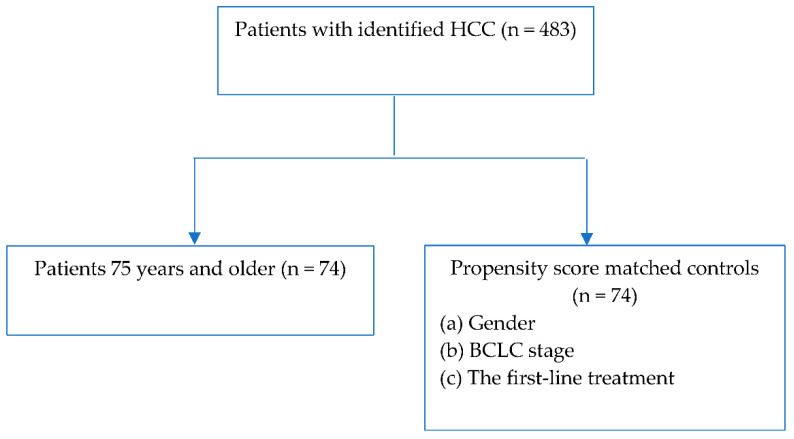
Flowchart.

The analyses included 148 patients (84 men, 57%) with HCC. Controls (<75 years) were matched to cases (≥75 years) according to gender, BCLC stage, and the first-line treatment (see Figure 1).

There were no differences between cases and controls in these examples, and no other baseline characteristics (Table 1).

**Figure 2:**
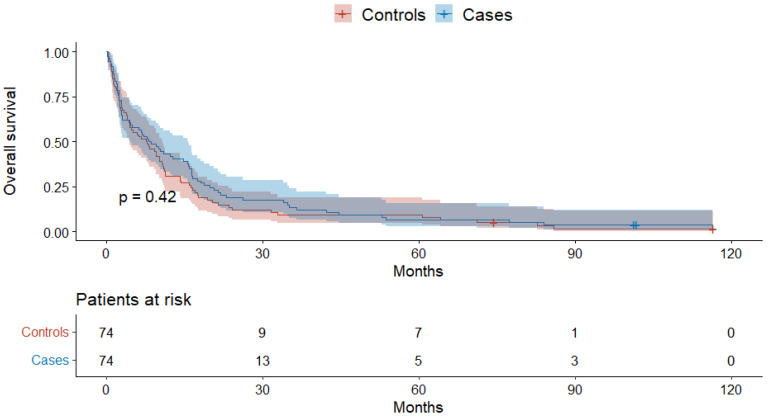
The overall survival of patients with HCC.

The overall survival in cases was comparable to controls (*p* = 0.42, Figure 2). The one-, two-, and three-year overall survival was 42% and 31%, 19% and 12%, and 12% and 9% in cases and controls, respectively (*p* = 0.2, 0.4, 0.8). Similarly, there was no difference in the one- and two-year progression-free survival: 28% and 18% vs. 10% and 7% in cases and controls, respectively (*p* = 0.2, 1).

There were several factors associated with the one- and two-year survival in geriatric patients with HCC: log (CRP), log (NLR), log (PLR), log (SII), and ALBI 3; although after controlling for the age and BCLC confounding, neither association remained significant. Similarly, in simple logistic regression analyses, log (CRP), log (NLR), log (PLR), and log (SII) were all associated with the three-year survival. However, this time, log (NLR) remained associated even after controlling for the age and BCLC confounding (OR 5.32, 95% CI 1.43–28.85). Complete results of regression analyses are presented in the Supplementary Material.

**Figure 3:**
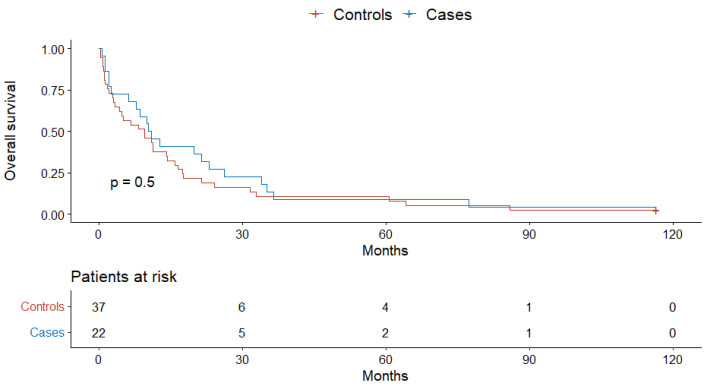
The overall survival of cases and controls with no comorbidities.

**Figure 4:**
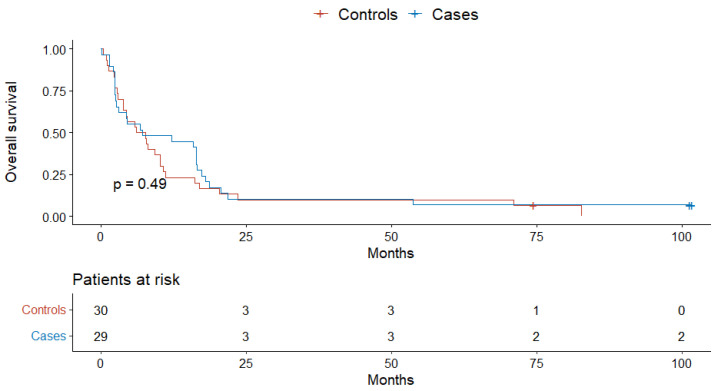
The overall survival of cases and controls with one or two comorbidities.

**Figure 5:**
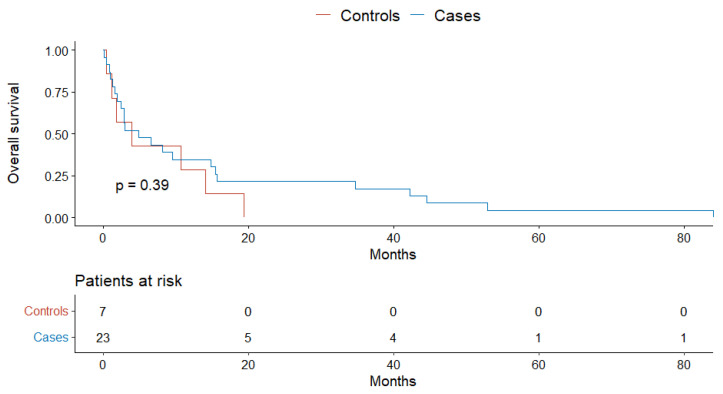
The overall survival of cases and controls with three or more comorbidities.

Finally, there was no case–control difference in the overall survival in the subpopulation of patients with no known comorbidities (*p* = 0.5), one and two comorbidities (*p* = 0.49), and three or more comorbidities (*p* = 0.39) (Figure 3, Figure 4 and Figure 5).

## 5. Discussion

Hepatocellular carcinoma belongs to the group of malignant diseases of the gastrointestinal tract with increasing morbidity and mortality. With increasing numbers of cases mainly recorded in developed countries (the USA, Canada, the UK, and Taiwan) there is also an increase in the group of patients of geriatric age.

The definition of a geriatric patient is considered an issue in itself. Currently, the generally accepted age threshold over 65 years is already being shifted in practice to over 70 and 75 years [20,21,22,23,24,25,26].

Despite the gradually increasing age, the evidence of treatment efficacy remains at quite a low level due to its rare inclusion in clinical trials [27]. Geriatric patients have a higher number of comorbidities compared to the younger population. These comorbidities may limit the indication of anticancer treatment and its efficacy. Older patients more often report complications after interventions and surgical procedures and complications of anticancer therapy.

At geriatric age, the most impaired function is in the liver. Due to aging, the volume of liver parenchyma shrinks by 20–40%, the blood flow is reduced by 35–50%, and enzymatic activity of hepatocytes and cells of the immune system, especially dendritic cells, decreases [28].

Generally, these patients are considered fragile due to their comorbidities and impaired drug metabolism. Nevertheless, the data documenting efficacy and safety in this therapeutic subgroup are quite limited, and the current guidelines do not recommend any change in the therapeutic strategy. [29].

The aim of our study was to compare the outcomes of a heterogeneous group of geriatric patients with the control group. The results of overall survival and progression-free survival were comparable in patients of both age subgroups at the same BCLC stage, performance status, and identical treatment modality. This conclusion is supported by the results published in the study with an identical age cut-off for performing resection [30,31], radiofrequency ablation [32,33], transarterial chemoembolization [24,34,35], and use of sorafenib [36,37,38], lenvatinib [39], and ramucirumab [40]. Likewise, several studies did not show the difference in recurrence-free survival when performing resection [30,41], worsened recurrence rate when performing RFA [33], or progression-free survival in systemic therapy [37,39].

An important role in the process of carcinogenesis is also played by a chronic, subclinical, ongoing inflammation in the tumor microenvironment, which represents a dynamic component that promotes tumor growth, proliferation, neoangiogenesis, and metastatic spread [42]. A key role in suppressing the cellular response is played by tissue macrophages (Kupffer cells), monocyte-derived macrophages, regulatory T cells (T_reg_), and monocyte-derived tumor-associated macrophages (TAMs). Macrophage components polarized into the M2 phenotypic form produce IL-10 and TGF-β, and chemokines promote chemotaxis of Tregs and ineffective Th2 cell response, promoting neoangiogenesis and tissue remodalation via production of VEGF and EGF. Via PD-L1 expression, they disable the effector phase CD8+ of T-cell immune response and reduce the expression of MHC glycoproteins class II molecules, producing IL17, which leads to an increase in the neutrophil count in the peripheral blood [43,44].

An important role in the tumor microenvironment is held by platelets, which, by producing PDGF, may directly promote the growth of the tumor tissue; moreover, they produce a chain of proinflammatory cytokines (P-selectin, lL-1, IL-3, IL-3) and anti-inflammatory factors (TGF-β) [45]. The production of TGF-β leads to significant immunosuppression and a reduced lymphocyte count [46].

NLR, PLR, and SII have been described as effectively independent factors of overall survival in geriatric patients with high-grade gliomas [47], non-small cell lung cancer [48], esophageal cancer [49], gastric cancer [50,51], and glioblastoma [52]. There are only published results providing data of elderly patients with HCC. In our study, NLR and SII indexes have been evaluated as statistically significant predictors of 3-year survival after controlling for the influence of age and BCLC, while none of them were significant in evaluating 1- to 2-year survival. These data are in contrast with the results of Li et al. (2018), who evaluated SII as an effective predictor of 1-year survival in patients over 75 years of age; however, these data have purposely monitored the geriatric population and showed significant heterogeneity, considering miscellaneous histological subtypes [10]. According to the data presented by Zaour et al. (2019), the elevated values of AFP, NLR, and PLR were associated with higher mortality in geriatric patients with HCC who underwent resection [53]. On the other hand, PLR values are not associated with overall survival in some malignancies; for instance, pancreatic cancer [54].

Another discussed topic in the treatment of geriatric patients is the impact of comorbidities on patients’ overall survival. A higher number of comorbidities in geriatric patients is linked to poorer survival in geriatric patients with head and neck cancers [55]; they reduce the number of patients on adjuvant therapy in the treatment of colorectal cancer [56] and increase the number of deaths in patients with breast cancer in stage I.–III. [57]. In the case of hepatocellular carcinoma, the presence of cardiovascular and respiratory tract diseases worsens the overall survival of patients who have undergone curative RFA treatment [26].

The outcomes of our study point out comparable overall survival and progression-free survival in patients 75 years or older in comparison with a younger age group. The overall survival in our study population has not been affected by the number of comorbidities in the semi-quantitative division into three groups.

The results of our study were limited by the low number of included patients and the retrospective character of the study. Another limitation of the study is the heterogeneous etiology of the primary liver disease and different stages of HCC according to BCLC criteria in both geriatric and younger patients. Considering the retrospective study design, we were unable to compare the incidence of treatment side effects in both groups of patients with HCC.

## 6. Conclusions

In our study, we found that patients older than 75 years with HCC have overall survival and progression-free survival comparable to younger patients matched in age, BCLC stage, and first-line treatment. In patients older than 75 years, the NLR value was an independent predictor of 3-year survival. These findings allow us to state that geriatric patients at risk of developing HCC should have the same surveillance program as younger patients. In indicating the anticancer treatment, age should not be considered a limitation, but in treatment, it is necessary to take comorbidities into account. Furthermore, the treatment should be adequately monitored, focusing on the incidence of the adverse events. Due to the small number of treated geriatric HCC patients, further studies confirming our results are required.

## Figures and Tables

**Table 1:** Clinical and demographic characteristics of the HCC cohort.

	Overall, *n* = 148 ^1^	<75 years, *n* = 74 ^1^	≥75 years, *n* = 74 ^1^	*p*-Value ^2^
Age	74 (65, 78)	64 (61, 69)	78 (76, 82)	<0.001
Sex (male)	84 (57%)	42 (57%)	42 (57%)	>0.9
Etiology (the most common)				0.2
HCV	24 (16%)	15 (20%)	9 (12%)	
ALD	54 (36%)	29 (39%)	25 (34%)	
NAFLD	25 (17%)	12 (16%)	13 (18%)	
BCLC stage				>0.9
0-A	16 (11%)	8 (11%)	8 (11%)	
B	35 (24%)	18 (24%)	18 (24%)	
C	62 (42%)	31 (42%)	31 (42%)	
D	35 (24%)	17 (23%)	17 (23%)	
Child–Pugh				>0.9
A	70 (49%)	34 (48%)	36 (50%)	
B	61 (43%)	31 (44%)	30 (42%)	
C	12 (8.4%)	6 (8.5%)	6 (8.3%)	
MELD	6.26 (5.94, 6.64)	6.25 (5.92, 6.57)	6.30 (6.06, 6.75)	0.2
Number of lesions	1.00 (1.00, 4.00)	1.00 (1.00, 4.00)	1.00 (1.00, 4.00)	0.7
Diameter of the largest lesion	62 (35, 94)	56 (32, 92)	65 (40, 100)	0.2
AFP	27 (6, 1105)	21 (7, 589)	46 (6, 1624)	0.8
CRP	12 (4, 42)	12 (4, 60)	14 (3, 37)	0.3
NLR	3.6 (2.4, 5.7)	3.8 (2.4, 6.6)	3.5 (2.5, 5.4)	0.6
PLR	159 (102, 223)	159 (95, 223)	158 (109, 221)	>0.9
SII	641 (312, 1318)	529 (301, 1399)	682 (464, 1308)	0.4
ALBI	−1.90 (−2.39, −1.36)	−1.90 (−2.38, −1.31)	−1.90 (−2.47, −1.46)	0.7
Albumin	33 (28, 38)	33 (28, 38)	34 (28, 38)	0.7
Creatinine	81 (71, 105)	78 (69, 97)	87 (71, 112)	0.08
Total bilirubin	22 (14, 35)	22 (15, 38)	22 (13, 32)	0.5
Alanine aminotransferase	0.68 (0.47, 1.15)	0.68 (0.46, 1.10)	0.70 (0.48, 1.23)	0.6
Aspartate aminotransferase	1.07 (0.62, 2.05)	1.06 (0.69, 2.00)	1.07 (0.59, 2.14)	0.6
Gamma-glutamyl transferase	2.5 (1.3, 4.7)	2.4 (1.4, 4.7)	2.7 (1.2, 4.7)	0.5
Alkaline phosphatase	2.17 (1.72, 4.06)	2.35 (1.77, 4.23)	2.16 (1.65, 3.73)	0.3
First-line treatment				0.9
Resection	10 (6.8%)	5 (6.8%)	5 (6.8%)	
Radiofrequency ablation	2 (1.4%)	1 (1.4%)	1 (1.4%)	
Transarterial chemoembolization	32 (22%)	16 (22%)	16 (22%)	
Sorafenib	49 (33%)	25 (34%)	25 (34%)	
Best supportive care	53 (36%)	26 (35%)	26 (35%)	
Liver transplantation	2 (1.4%)	1 (1.4%)	1 (1.4%)	
ECOG Performance Status				0.5
0	5 (3.4%)	4 (5.4%)	1 (1.4%)	
1	106 (72%)	52 (70%)	54 (73%)	
2	37 (25%)	18 (24%)	19 (26%)	

^1^ Median (IQR); *n* (%). ^2^ Wilcoxon rank sum test; Pearson’s Chi-squared test; Fisher’s exact test. HCV—hepatitis C virus, ALD—alcoholic liver disease, NAFLD—non-alcoholic fatty liver disease, BCLC—Barcelona Clinic Liver cancer, MELD—Model for End-Stage Liver Disease, AFP—Alpha-Fetoprotein, CRP—C-reactive protein, NLR—Neutrophil-to-lymphocyte ratio, PLR—platelet-to-lymphocyte ratio, SII—systemic immune-inflammation index, ALBI—albumin-bilirubin ratio, ECOG—Eastern Cooperative Oncology Group.

## Data Availability

The data used to support the findings of this study are available from the corresponding author upon request.

## Supplementary Materials

The following supporting information can be downloaded at: https://www.mdpi.com/article/10.3390/jcm11144183/s1. Table S1. Association of baseline factors and one-year overall survival—simple analysis. Table S2. Association of baseline factors and one-year overall survival—multivariant analysis adjusted for age and BCLC. Table S3. Association of baseline factors and two-year overall survival—simple analysis. Table S4. Association of baseline factors and two-year overall survival—multivariant analysis adjusted for age and BCLC. Table S5. Association of baseline factors and three-year overall survival—simple analysis. Table S6. Association of baseline factors and three-year overall survival—multivariant analysis adjusted for age and BCLC.
