# Supplementary material for: Inflammatory Indexes as Prognostic Factors of Survival in Geriatric Patients with Hepatocellular Carcinoma: A Case Control Study of Eight Slovak Centers
Source: J Clin Med. 2022 Jul 19;11(14):4183. doi: 10.3390/jcm11144183 (PMC9318669; doi:10.3390/jcm11144183)
Supplement: Supplementary file 1 [file jcm-11-04183-s001.zip › jcm-1779346-supplementary.pdf]

Article

Inflammatory Indexes as Prognostic Factors of Survival in Geriatric Patients with Hepatocellular Carcinoma: A Case Control Study of Eight Slovak Centers

Dominik Safcak [1], Sylvia Drazilova [2,3,*], Jakub Gazda [3], Igor Andrasina [1], Svetlana Adamcova-Selcanova [4], Lea Balazova [1], Radovan Barila [5], Michal Mego [6], Marek Rac [7], Lubomir Skladany [4], Miroslav Zigrai [8], Martin Janicko [3] and Peter Jarcuska [3]

1 Department of Radiotherapy and Oncology, East Slovakia Institute of Oncology, Rastislavova 43, 041 91 Kosice, Slovakia; safcak@vou.sk (D.S.); andrasina@vou.sk (I.A.); lea.balazova@gmail.com (L.B.)
2 Internal Medicine Department, Hospital Poprad a.s., Banicka 803, 058 01 Poprad, Slovakia
3 2nd Department of Internal Medicine, P. J. Safarik University, Faculty of Medicine and L. Pasteur University Hospital, Trieda SNP 1, 040 11 Kosice, Slovakia; jakub.gazda@upjs.sk (J.G.); martin.janicko@upjs.sk (M.J.); peter.jarcuska@upjs.sk (P.J.)
4 2nd Department of Internal Medicine, HEGITO, F. D. Roosevelt University Hospital, Namestie L Svobodu 1, 975 17 Banska Bystrica, Slovakia; sselcanova@gmail.com (S.A.-S.); lubomir.skladany@gmail.com (L.S.)
5 Oncological Cluster, Stefan Kukura Hospital in Michalovce, Spitalska Ulica 2, 071 01 Michalovce, Slovakia; radovan.barila@gmail.com
6 2nd Department of Oncology, Faculty of Medicine, Comenius University and National Oncology Institute of Slovakia, Klenova 1, 833 10 Bratislava, Slovakia; misomego@gmail.com
7 Department of Internal Medicine, Teaching Hospital Nitra, Spitalska 6, 949 01 Nitra, Slovakia; marek.rac@fnnitra.sk
8 1st Department of Internal Medicine, Ladislav Derer University Hospital in Bratislava, Limbova 5, 833 05 Bratislava-Kramare, Slovakia; miroslav.zigrai@szu.sk
* Correspondence: sylvia.drazilova@upjs.sk

Citation: Safcak, D.; Drazilova, S.; Gazda, J.; Andrasina, I.; Adamcova-Selcanova, S.; Balazova, L.; Barila, R.; Mego, M.; Rac, M.; Skladany, L.; et al. Inflammatory Indexes as Prognostic Factors of Survival in Geriatric Patients with Hepatocellular Carcinoma: A Case Control Study of Eight Slovak Centers. *J. Clin. Med.* **2022**, *11*, 4183. https://doi.org/10.3390/jcm11144183

Academic Editor: Gerardo Rosati

Received: 3 June 2022
Accepted: 14 July 2022
Published: 19 July 2022

Publisher's Note: MDPI stays neutral with regard to jurisdictional claims in published maps and institutional affiliations.

Copyright: © 2022 by the authors. Licensee MDPI, Basel, Switzerland. This article is an open access article distributed under the terms and conditions of the Creative Commons Attribution (CC BY) license (https://creativecommons.org/licenses/by/4.0/).

Abstract: Background and Aims: Hepatocellular cancer (HCC) often occurs in geriatric patients. The aim of our study was to compare overall survival and progression-free survival between geriatric patients (>75 years) and patients younger than 75 years and to identify predictive factors of survival in geriatric patients with HCC. Material and Methods: We performed a retrospective analysis of patients with HCC diagnosed in Slovakia between 2010–2016. Cases (HCC patients ≥75 years) were matched to controls (HCC patients <74 years) based on the propensity score (gender, BCLC stage and the first-line treatment). Results: We included 148 patients (84 men, 57%) with HCC. There were no differences between cases and controls in the baseline characteristics. The overall survival in geriatric patients with HCC was comparable to younger controls ($p = 0.42$). The one-, two-, and three-year overall survival was 42% and 31%, 19% and 12%, and 12% and 9% in geriatric patients and controls, respectively (p = 0.2, 0.4, 0.8). Similarly, there was no difference in the one- and two-year progression-free survival: 28% and 18% vs. 10% and 7% in geriatric HCC patients and controls, respectively (p = 0.2, 1, -). There was no case–control difference between geriatric HCC patients and younger HCC controls in the overall survival in the subpopulation of patients with no known comorbidities ($p = 0.5$), one and two comorbidities ($p = 0.49$), and three or more comorbidities ($p = 0.39$). Log (CRP), log (NLR), log (PLR), and log (SII) were all associated with the three-year survival in geriatric HCC patients in simple logistic regression analyses. However, this time, only log (NLR) remained associated even after controlling for the age and BCLC confounding (OR 5.32, 95% CI 1.43–28.85). Conclusions. We found no differences in overall survival and progression-free survival between older and younger HCC patients. Parameters of subclinical inflammation predict prognosis in geriatric patients with HCC. A limitation of the study is small number of the treated patients; therefore, further investigation is warranted.

Keywords: hepatocellular cancer; geriatric patients; overall survival; progression-free survival

Supplementary Materials:

Table S1 Association of baseline factors and one-year overall survival - simple analysis

**One-Year Survival**	OR	95%CI lower	95%CI higher	P
Age	0.99	0.95	1.02	0.40
Sex (male-reference)	0.59	0.29	1.17	0.14
Child-Pugh score	1.79	1.38	2.42	<0.001
Child-Pugh A	Ref	Ref	Ref	
Child-Pugh B	4.92	2.32	10.99	<0.001
Child-Pugh C	14.67	2.64	275.45	0.01
BCLC 0-A	Ref	Ref	Ref	
BCLC B	1.20	0.32	5.09	0.79
BCLC C	9.40	2.82	37.82	<0.001
BCLC D	49.50	9.59	410.98	<0.001
Log(CRP)	1.66	1.25	2.27	<0.001
Log(NLR)	1.95	1.17	3.45	0.02
Log(PLR)	2.48	1.29	5.05	0.009
Log(SII)	1.90	1.28	2.92	0.002
ALBI 1	Ref	Ref	Ref	
ALBI 2	1.76	0.71	4.43	0.22
ALBI 3	6.79	2.18	23.52	0.001

Table S2 Association of baseline factors and one-year overall survival - multivariant analysis adjusted for age and BCLC

**One-Year Survival Adjusted for Age and BCLC**	OR	95%CI lower	95%CI higher	P
Log(CRP)	1.10	0.75	1.61	0.62
Log(NLR)	1.52	0.73	3.30	0.27
Log(PLR)	1.69	0.73	4.08	0.22
Log(SII)	1.58	0.92	2.79	0.10
ALBI 1	Ref	Ref	Ref	
ALBI 2	1.11	0.38	3.24	0.85
ALBI 3	2.40	0.58	10.47	0.23

Table S3 Association of baseline factors and two-year overall survival - simple analysis

**Two-Years Survival**	OR	95%CI lower	95%CI higher	P
Age	0.98	0.94	1.03	0.48
Sex (male-reference)	0.41	0.14	1.05	0.08
Child-Pugh score	1.53	1.11	2.27	0.02
Child-Pugh A	Ref	Ref	Ref	
Child-Pugh B	3.59	1.32	11.55	0.02
Child-Pugh C	3.53	0.62	66.85	0.24
BCLC 0-A	Ref	Ref	Ref	

BCLC B	3.21	0.95	11.44	0.06
BCLC C	25.29	6.06	137.10	<0.001
BCLC D	43.71	6.73	876.95	<0.001
Log(CRP)	1.64	1.15	2.46	0.009
Log(NLR)	2.70	1.35	6.04	0.008
Log(PLR)	2.32	1.01	5.62	0.05
Log(SII)	2.03	1.23	3.54	0.008
ALBI 1	Ref	Ref	Ref	
ALBI 2	1.51	0.48	4.34	0.46
ALBI 3	3.68	0.87	19.05	0.09

Table S4 Association of baseline factors and two-year overall survival - multivariant analysis adjusted for age and BCLC

**Two-Years Survival Adjusted for Age and BCLC**	OR	95%CI lower	95%CI higher	P
Log(CRP)	1.24	0.79	1.99	0.36
Log(NLR)	2.58	1.01	7.75	0.06
Log(PLR)	2.35	0.68	8.42	0.18
Log(SII)	2.00	0.94	4.63	0.08
ALBI 1	Ref	Ref	Ref	
ALBI 2	1.05	0.29	3.58	0.94
ALBI 3	1.54	0.27	10.05	0.63

Table S5 Association of baseline factors and three-year overall survival - simple analysis

**Three-Years Survival**	OR	95%CI lower	95%CI higher	P
Age	0.98	0.92	1.03	0.49
Sex (male-reference)	0.27	0.06	0.88	0.05
Child-Pugh score	1.52	1.05	2.44	0.05
Child-Pugh A	Ref	Ref	Ref	
Child-Pugh B	2.95	0.96	11.04	0.07
Child-Pugh C	2.39	-	-	0.99
BCLC 0-A	Ref	Ref	Ref	
BCLC B	2.63	7.37	9.51	0.13
BCLC C	4.74	7.30	943.84	<0.001
BCLC D	2.45	-	-	0.99
Log(CRP)	2.12	1.36	3.62	0.002
Log(NLR)	3.09	1.48	7.41	0.005
Log(PLR)	2.64	1.10	6.74	0.03
Log(SII)	2.20	1.29	3.99	0.006
ALBI 1	Ref	Ref	Ref	
ALBI 2	1.35	0.34	4.52	0.64
ALBI 3	3.43	0.62	26.28	0.18

Table S6 Association of baseline factors and three-year overall survival - multivariant analysis adjusted for age and BCLC

**Three-Years Survival Adjusted for Age and BCLC**	OR	95%CI lower	95%CI higher	P
Log(CRP)	1.62	0.96	2.96	0.09
Log(NLR)	5.32	1.43	28.85	0.03
Log(PLR)	2.82	0.74	11.58	0.13
Log(SII)	1.11	0.53	2.08	0.04
ALBI 1	Ref	Ref	Ref	
ALBI 2	0.86	0.19	3.47	0.84
ALBI 3	1.06	0.14	10.08	0.95
